# An orthotropic continuum model with substructure evolution for describing bone remodeling: an interpretation of the primary mechanism behind Wolff’s law

**DOI:** 10.1007/s10237-023-01755-w

**Published:** 2023-08-05

**Authors:** Ivan Giorgio, Francesco dell’Isola, Ugo Andreaus, Anil Misra

**Affiliations:** 1 Department of Civil, Construction-Architectural and Environmental Engineering (DICEAA), University of L’Aquila, 1, P.zza Ernesto Pontieri, Monteluco di Roio, L’Aquila, 67100 Italy; 2 International Research Center for the Mathematics and Mechanics of Complex Systems (M &MoCS), University of L’Aquila, 1, P.zza Ernesto Pontieri, Monteluco di Roio, L’Aquila, 67100 Italy; 3 Department of Structural and Geotechnical Engineering (DISG), Università di Roma La Sapienza, 18, Via Eudossiana, Rome, 00184 Italy; 4 Civil, Environmental and Architectural Engineering Department (CEAE), The University of Kansas, 1530 W. 15th Street, Learned Hall, Lawrence, 66045–7609 Kansas USA; 5 Faculty of Mechanical and Industrial Engineering, Warsaw University of Technology, ul. Narbutta 85, Warsaw, 02-524 Poland; 6 CNRS Fellow, ENS Paris-Saclay, 4, avenue des Sciences, Gif-sur-Yvette, 91190 France

**Keywords:** Bone functional adaptation, Growth/resorption processes, Bone remodeling, Orthotropic constitutive law, Variational formulation

## Abstract

We propose a variational approach that employs a generalized principle of virtual work to estimate both the mechanical response and the changes in living bone tissue during the remodeling process. This approach provides an explanation for the adaptive regulation of the bone substructure in the context of orthotropic material symmetry. We specifically focus upon the crucial gradual adjustment of bone tissue as a structural material that adapts its mechanical features, such as materials stiffnesses and microstructure, in response to the evolving loading conditions. We postulate that the evolution process relies on a feedback mechanism involving multiple stimulus signals. The mechanical and remodeling behavior of bone tissue is clearly a complex process that is difficult to describe within the framework of classical continuum theories. For this reason, a generalized continuum elastic theory is employed as a proper mathematical context for an adequate description of the examined phenomenon. To simplify the investigation, we considered a two-dimensional problem. Numerical simulations have been performed to illustrate bone evolution in a few significant cases: the bending of a rectangular cantilever plate and a three-point flexure test. The results are encouraging because they can replicate the optimization process observed in bone remodeling. The proposed model provides a likely distribution of stiffnesses and accurately represents the arrangement of trabeculae macroscopically described by the orthotropic symmetry directions, as supported by experimental evidence from the trajectorial theory.

## Introduction

Wolff’s law statement concerns the concept of “bone functional adaptation” to mechanical loadings. It is a well-known principle named after the German anatomist and surgeon Julius Wolff which states that *the micro-mechano-morphological properties of bone are primarily, albeit not entirely, a consequence of bone response to mechanical deformation * (Wolff 1892)*.* More specifically, the law asserts that bone will remodel itself over time in response to the mechanical loads placed upon it, becoming denser and stronger in areas subjected to increased stress while weakened and more susceptible to fractures in areas subjected to less stress. Wolff’s law has important implications for the prevention and treatment of conditions such as osteoporosis, as weight-bearing exercise and other forms of physical activity that can help maintain bone density and reduce the risk of fractures. Furthermore, specific dental treatments utilize Wolff’s law to enhance the alignment of teeth within the dental arches (Cornelis et al. 2021). Bone remodeling in the vicinity of osteomyelitis (Masters et al. 2019) affected bone could also be influenced by the changed local loading environment both during disease development and after surgical treatment (Lamm et al. 2015).

This *remodeling process* is ensured by the synergic activity of three types of bone cells with different functions in the growth, maintenance, and repair of bone tissue: *osteoclasts*, *osteoblasts*, and *osteocytes*. Osteoclasts are large, multinucleated cells that dismantle mineral tissue through a process called *resorption*. They are responsible for removing old, damaged bone tissue and releasing calcium and other minerals into the bloodstream. Osteoblasts are cells that synthesize and deposit new bone tissue through a process called *ossification*. They secrete collagen and other proteins that form the framework for new bone tissue and also help to mineralize the bone matrix with hydroxyapatite, i.e., a calcium phosphate mineral. Osteoblasts are responsible for the growth and repair of bone tissue and play a key role in maintaining bone density and strength. Osteocytes are mature bone cells, deriving from the differentiation of osteoblasts, embedded within the mineralized matrix of bone tissue buried into tiny spaces called lacunae. They play a crucial role in regulating bone metabolism and responding to mechanical stresses placed on the bone that involves a process called *mechanotransduction*. Osteocytes are a kind of sensor cell connected to one another and to the bone surface by long, branching structures called dendritic processes housed in an intricate network of canals between the lacunae, called canaliculi, and filled with interstitial (periosteocytic) fluid. They communicate with the other bone cells to coordinate bone remodeling and repair. The activity of all these cells is essential for maintaining a balance between bone resorption and bone formation and for ensuring that bone tissue is constantly being renewed, replaced, and fit for its mechanical task.

The activity of bone cells results in the constitution and continuous shaping of two types of bone tissue that can be found in the human body: *cortical* and *trabecular* bone. They have different characteristics and serve different functions. Cortical bone, also known as compact bone, is the denser, more resistant outer layer of bone that makes up the shafts of long bones, such as the femur. It is composed of tightly packed layers of mineralized collagen fibers and is responsible for providing structural support and protection for the body and vital organs. They form cylindrical substructures called osteons. Cortical bone, due to its mechanical properties and space distribution, is relatively resistant to bending and twisting forces and is well-suited for weight-bearing activities. Trabecular bone, also known as spongy bone or cancellous bone, is a lighter, less dense type of bone tissue that is found at the ends of long bones and in the interior of short bones. It is composed of a network of bony struts called trabeculae that provide support for the bone and help distribute weight and stress. Trabecular bone is more compliant and can better withstand compression forces, making it an essential component of the body’s shock-absorbing system, also considering that it is filled with bone marrow that has damping properties. Both types of bone tissue are important for maintaining overall bone health and function (Giorgio et al. 2021).

The bottom line is that the structure of bone tissue maintains a delicate balance between providing strength and support to the body while minimizing the cost of nutrients and required energy for the living cells operating in it (Bednarczyk and Lekszycki 2016, 2022). By adjusting the distribution of bone mass and arranging its microstructure in an efficient composite structure, bone tissue is able to achieve this balance and maintain its function and integrity over time. Bone achieves this balance in response to the mechanical strain induced by external loads to which it is subjected. This means that areas subjected to more significant stress, such as the weight-bearing bones in the legs, will have more mass density than areas subjected to less stress, such as the bones in the fingers. This can be achieved, for example, through changes in the diameters and numbers of the trabeculae (Zhao et al. 2018). Besides, bone can also achieve an efficient composite structure by re-arranging its microstructure to maximize strength while minimizing weight, specifically by reorienting the pattern of the trabeculae depending on the direction of the external forces applied (Kivell 2016).

Usually, to explain the reorientation of the inner micro-architecture of bone tissue, the so-called *trajectorial theory* (Lanyon 1974) is employed, which assumes that the principal stresses are one of the main factors in determining the structure of trabecular bone tissue. More precisely, it is observed that the directions of the cancellous microstructure tend to be aligned to the eigenvectors of stress. Observed discrepancies in this alignment is mainly due to the different kinds of external loads that actually are applied to the bone tissue in different situations: each of these loads obviously determine different directions of principal stresses. Therefore, the effective trabecular orientation has to be considered as the weighted average result produced by the several mechanical loads applied during the functioning of the system “bone structure”.

From a modeling viewpoint, bone remodeling can be regarded as a feedback phenomenon in the control theory framework (Frost 1987; Turner 1991). The stiffness tensor and material strength can be considered controlled quantities in this biological process. Thus, a control loop is established to regulate those variables at a setpoint, i.e., the so-called homeostatic state. To this end, the amount and the distribution of bone mass are changed to guarantee optimal functionality of the bone tissue. Indeed, based on the external request for mechanical resistance, the active bone cells, i.e., process actuators, can resorb or synthesize bone tissue based on the information provided by the osteocytes in charge of sensing the mechanical state of the tissue. In an effort to explain this biological process, an early attempt has been made introducing an evolutionary equation for apparent mass density (bone tissue is a porous material) that relied on a mechanical feedback signal (Beaupré et al. 1990; Mullender and Huiskes 1995). This is because bone mass density is directly linked to its stiffness; therefore, any changes in mass density would also alter the elastic modulus. Based on empirical evidence, this relationship between apparent mass density and stiffness has been introduced phenomenologically (Carter and Hayes 1976; Currey 1988). Thus, the change in the mass density also results in an adaptive evolution of the elastic modulus that optimizes the mechanical response of the bone. This approach is particularly suited for an isotropic behavior of the considered tissue since the apparent mass density is a scalar quantity and, hence, does not directly imply any mechanical properties linked with the reorientation of the trabecular microstructure. Therefore, the initial models incorporating mass density evolution have been primarily utilized for isotropic materials.

To take into account also the reorientation evolution, thus, it has been proposed a direct evolution of the stiffness tensor through a remodeling tensor that, in this context, is related to variables such as the apparent mass density and fabric tensor, which are intricately linked to the porosity and directionality of the trabeculae (Cowin et al. 1992; Doblaré and Garcıa 2002). These formulations can also be applied to orthotropic or anisotropic materials because of the tensorial nature of the stiffness tensor. However, some approaches are still based on mass density, even in orthotropic scenarios. Using phenomenological relationships, the distinct elastic moduli can be evaluated from the mass density. One potential limitation of this proposal is that the orthotropy directions are predetermined. In Sarikanat and Yildiz (2011), these directions are postulated to be aligned with the stress isolines following the trajectorial theory. However, it is worth noting that this perspective does not entail any form of evolution for the orthotropy directions.

The feedback signal is usually evaluated using a mechanical stimulus that conveys the information of the mechanical state considered pertinent for the particular adopted formulation. This mechanical state can be specified by strain energy density (Huiskes et al. 1987), effective stress (Carter et al. 1987; Beaupré et al. 1990), strain peak (Turner 1998), or damage variable (Prendergast and Taylor 1994; Hambli 2014), to name a few. There are two types of stimulus formulation: local and nonlocal. Local models apply the evolution law to each point of the body individually without directly considering the mechanical state of nearby points. Conversely, nonlocal models provide an evolution for each point in the body incorporating data from that point and other points in a specified finite surrounding volume of material. Nonlocal models are based on convolution integrals (Lekszycki and dell’Isola 2012; Kumar et al. 2011; George et al. 2018, 2019) or diffusion equations (Giorgio et al. 2019; Scerrato et al. 2022) and guarantee the possibility of describing the interaction between bone tissue and graft of bio-resorbable material or between healthy bone and necrotic tissue in which osteocytes could be dead. This kind of interaction is prevented from happening in local models because no stimulus can be generated where osteocytes are absent. On the contrary, with the nonlocal effect, the stimulus originating in surrounding areas can reach zones without osteocytes and trigger the remodeling process, as in fact occurs.

Bone tissue is characterized by a variegated architecture with multiple levels of complexity. Due to this, the conventional continuum elastic theory fails to accurately describe its intricate mechanics in different circumstances. For this reason, generalized continuum theories can be adopted to imitate the behavior of the real tissue more accurately and efficiently. The presence of high contrast in the mechanical properties of the bone may lead to the use of second gradient models that can represent a more rich behavior in terms of deformation modes and boundary conditions sustainable (Fedele 2022; dell’Isola et al. 2022; Solyaev et al. 2020; Vazic et al. 2021, 2023; Sarar et al. 2023; Carter et al. 1987; Andres et al. 2001). Strictly related models are those that deal with fiber-reinforced composites (Steigmann 2012; Franciosi et al. 2019; Spagnuolo 2022) since the mineralization of the tissue starts from a network of collagen fibers in the new woven bone.

Another aspect that is essential in a refined description of bone tissue is porosity. Models that take into account this aspect can explain phenomena involving inner pore pressure and possible dissipation due to the interaction between the solid matrix and viscous fluids, namely bone marrow, interstitial fluid, and blood, contained in it. The dissipation can be introduced using phenomenological laws, as can be found in (Jankowski et al. 2022; Sessa 2023), or more fundamentally, using Rayleigh functional (Giorgio et al. 2016). Due to the various types of porosity found in bone tissue, it is possible to utilize models with double or even higher porosity (see, e.g., De Cicco and De Angelis 2020; De Cicco 2022). Naturally, it is possible to use more complex models, such as poroelastic materials with fiber reinforcement and potential fluid inclusion, to depict the mechanical behavior of bones accurately (Tomic et al. 2014; Grillo et al. 2015, 2018; Cuomo et al. 2022; Gazzo et al. 2020).

Experimental evidence on bone samples revealed nonclassical effects associated with the microstructure (Park and Lakes 1986). This exotic behavior can be considered in the context of micropolar theory (see, e.g., Eremeyev and Pietraszkiewicz 2012, 2016; Eremeyev et al. 2017, 2020; La Valle 2022).

The implementation of a variational formulation represents an elegant and effective approach to comprehending the relationship between the evolution of bone micro-architecture and the underlying physical laws that govern their behavior. This technique proves particularly advantageous in addressing complex problems, such as those encountered in studying bones and growth phenomena (Grillo and Di Stefano 2023; Grillo and Di Stefano 2023). This powerful approach can also be used to address challenging problems, such as those involving damage, that are intrinsically connected to the evolution of bone tissue (Placidi et al. 2018, 2018, 2019; Timofeev et al. 2021; De Angelis 2007). Numerous authors interpret the remodeling of bone tissue as a complex optimization problem in which the local properties of mass and stiffness are regarded as conflicting objectives that must be balanced. From this perspective, once again, the variational tools provide a powerful arsenal at our disposal to obtain the optimized substructure underlying the bone tissue (see, as general reference Bendsoe and Sigmund 2003; Eschenauer and Olhoff 2001, and as a more specific application to the bone Nowak 2010, 2020).

In this paper, we propose a possible way to incorporate the fundamental aspect of reorientating the trabecular microarchitecture of bone tissue in an elastic continuum scenario at a macroscopic level of observation. The material behavior is assumed to be orthotopic. The governing equation for the remodeling process is deduced from a variational point of view through the definition of a generalized virtual work that takes into account both the mechanical response and the evolution of the trabecular microstructure. The stimuli used as feedback signals to guide the process are defined on an energetic basis and have a nonlocal formulation that relays on diffusion equations.

## Modeling of the evolution process related to bone functional adaptation

### Kinematics

As happens in standard continuum mechanics, the position $\varvec{x}$ occupied by any particle $\varvec{X}$ at the time $t\in [t_{0}, t_{f}]$ in the *current configuration* is given by the placement map $\varvec{\chi }:{\mathcal {B}}^{*}\times [t_{0}, t_{f}],\rightarrow {\mathcal {E}}^{\textrm{3}}$, where ${\mathcal {E}}^{\textrm{3}}$ is the Euclidean 3D affine space,

1 $$\begin{aligned} \varvec{x}=\varvec{\chi }(\varvec{X},t) \end{aligned}$$

where the material particles in the *reference configuration* are denoted by $\varvec{X}$ in the domain ${\mathcal {B}}^{*}$.

Aiming to describe the bio-mechanical behavior of a bone tissue as a continuum deformable body having orthotropic material symmetry, in each material point and at every time instant, we introduce a material symmetry rotation $\varvec{Q}(X,t).$

Equivalently, one may consider the three time-variable orthogonal unit vectors

$$\begin{aligned} \varvec{Q}(X,t)\varvec{e}_{i}:=\varvec{A}_{i}(X,t), \end{aligned}$$

where $\left\{ \varvec{e}{}_{i}\right\}$ is an orthonormal basis in the space of displacements of ${\mathcal {E}}^{\textrm{3}}$. Moreover, we assume that the organism in which the bone tissue is incorporated has a bone tissue control system activated by suitable *stimuli, *which are generated as a response of the tissue mechanical state and which determine its production and adsorption.

Therefore, to model the bio-mechanical evolution of the bone tissue, we use the following list of kinematical descriptors:

1. The displacement field, denoted by $\varvec{u}(\varvec{X},t)=\varvec{\chi }(\varvec{X},t)-\varvec{X},$
2. The angles characterizing the rotation $\varvec{Q}$ with respect to an Eulerian basis,
3. The set of material parameters, which characterize the orthotropic quadratic elastic energy,
4. The set of stimuli that are needed to guide the remodeling process.

The spirit here is to consider a generalized continuum theory where the overall evolution of the system is given by the standard descriptor, i.e., the placement $\varvec{\chi }$ or, equivalently, the displacement $\varvec{u}$, as well as further ones that specify both the mechanical properties of the tissue and the process of the bio-mechanical transduction guided on the information-based stimuli production.

Let us denote by $\varvec{\psi }$, the variables specifying the orientation $\varvec{A}_{1}(X,t)$ and $\varvec{A}_{2}(X,t)$, by $\varvec{p}(\varvec{X},t)$ the entire set of elastic moduli, and, finally, by $\varvec{S}=\{S_{p_{i}}\}$ the vector whose components represent the bio-mechanical stimuli, being each of them associated with each modulus $p_{i}$.

In the present model, the deformation measure is given in terms of the deformation gradient tensor $\varvec{F}=\nabla \varvec{\chi }$, and specifically by means of the linearized strain tensor

2 $$\begin{aligned} \varvec{E}=\frac{1}{2}\left( \varvec{F}^{\top } +\varvec{F}\right) -{\textbf{I}}=\frac{1}{2} \left( \nabla \varvec{u}^{\top }+\nabla \varvec{u}\right) \end{aligned}$$

Because of the complexity of the system, we presently confine our further development to the particular significant case in which a bi-dimensional domain ${\mathcal {B}}^{*}$ is taken into account. The interesting results we believe to have obtained will motivate the development of a full 3D model. In the case of 2D evolutions, as only one angle is sufficient to characterize the orientation $\varvec{Q}$ defining the evolutionary reorientation of the trabecular substructure, we can proceed as follows. We introduce the macro-rotation related to the polar decomposition of $\varvec{F}$

3 $$\begin{aligned} \varvec{F}=\varvec{R}(\vartheta )\,\varvec{U} \end{aligned}$$

in which the rotation $\varvec{R}$ is characterized by an angle $\vartheta$ in the bi-dimensional problem and $\varvec{U}$ gives the deformation. Then, assuming that the microstructure associated with the trabeculae can be represented by the angle $\psi$, which gives, e.g., the orientation of $\varvec{A}_{1}$, we can introduce the relative angle

4 $$\begin{aligned} \gamma (\varvec{X},t)=\vartheta (\varvec{X},t)-\psi (\varvec{X},t). \end{aligned}$$

that represents a measure of misalignment between the material directions of symmetry and the current orientation of the infinitesimal material cube at the same location due to the application of the external load.

### Principle of virtual work

A generalized virtual-work principle is postulated for the independent virtual displacements $\delta \varvec{u}$, its virtual gradient $\delta \nabla \varvec{u}$, the virtual change in the parameters $\delta \gamma$, and $\delta p_{i}$ as follows:

5 $$\begin{aligned}&\int _{{\mathcal {B}}^{*}}\delta w_{m}\,\text {d}\omega -\int _{\partial {\mathcal {B}}^{*}}\varvec{f} \cdot \delta \varvec{u}\,\text {d}l-\int _{\partial {\mathcal {B}}^{*}} \mathbb {F}\cdot \delta \nabla \varvec{u}\cdot \varvec{n}\,\text {d}l\nonumber \\&\quad +\int _{{\mathcal {B}}^{*}}\,(c_{\gamma }{\dot{\gamma }} +\tau _{\gamma }\gamma )\delta \gamma \,\text {d}\omega \nonumber \\&\quad +\sum _{i}\int _{{\mathcal {B}}^{*}}\,\left[ c_{p_{i}}\dot{p_{i}} -{\mathscr {A}}(S_{p_{i}})\right] \delta p_{i}\,\text {d}\omega =0 \end{aligned}$$

The first line of (5) is representative of the mechanical contribution given by the stored strain energy, namely $w_{m}$, and the external virtual work done by the traction $\varvec{f}$ per unit line and the density of double force $\mathbb {F}$ since we consider second gradient materials (Madeo et al. 2012; Giorgio et al. 2017; Ganghoffer et al. 2019). The last two contributions are related to the mechano-biological evolution driven by the mechanical response. In them, we can interpret $c_{\gamma }{\dot{\gamma }}$ and $\tau _{\gamma }\gamma$ as *remodeling couples*, where $c_{\gamma }$ and $\tau _{\gamma }$ are constitutive parameters that relate to the time rate of the evolution of $\gamma$ and the evolution of $\gamma$, respectively. In addition, $c_{p_{i}}\dot{p_{i}}$ and $-{\mathscr {A}}(S_{p_{i}})$ could be thought as *remodeling actions* that expend a mechano-biological work on the material parameter $p_{i}$. Similarly to the previous term, $c_{p_{i}}$ is a constitutive parameter linked to the time rate of the evolution of the elastic modulus $p_{i}$ and ${\mathscr {A}}$ is a biological potential associated with the evolution of $p_{i}$. It is worth noting that the weak contribution related to the remodeling actions in the last term of (5) produces an evolutionary term very similar to the one used in Eq. (5) in Giorgio et al. (2019).

The key idea of the model is to define a total bio-mechanical work as the sum of two contributions: 1) the first one able to characterize the mechanical response of bone and 2) the second one to determine the evolution of the inner architecture of the tissue considering both the orientation of the substructure and its stiffness. Since the complex phenomena involving the bone tissue develop on a multilevel time scale and the remodeling process is happening very slowly, herein, we neglect the rapid dynamic associated with the bone mechanics assuming a clear separation of time scale between the fastest variations related to the application of mechanical loads and the slow process of the remodeling. For this aim, we consider loads slowly variable in time, and thus, we neglect inertial phenomena.

To be more specific, we can introduce the energy (Cyron and Aydin 2017; DiCarlo and Quiligotti 2002):

6 $$\begin{aligned} w(\varvec{F},\nabla \varvec{F},\psi ,\gamma , \varvec{p},\varvec{S})=w_{m}(\varvec{F}, \nabla \varvec{F},\psi ,\varvec{p}) +w_{r}(\gamma ,\varvec{p},\varvec{S}) \end{aligned}$$

where $w_{m}$ is the standard orthotropic second gradient mechanical deformation energy and $w_{r}$ is the mechano-biological term which captures biologically induced changes of the bone substructure and, thus, is representative of the stress-free configuration of the tissue over the remodeling process.

To describe the mechanical response of the bone tissue, we take as our starting point the constitutive equations for orthotropic materials. In this paper, we adopt, for the strain energy density $w_{m}$, the Galilean invariant function:

7 $$\begin{aligned} w_{m}(\varvec{E},\nabla \varvec{E}) =w_{1}(\varvec{E})+w_{2}(\nabla \varvec{E}) \end{aligned}$$

in which, for simplicity, we consider two additive terms: one related to a first gradient behavior and the other to a second gradient one. Particularly, the first gradient contribution can be specialized as

8 $$\begin{aligned} w_{1}=\hat{w_{1}}(J_{1},J_{2},J_{4},J_{5},J_{6},J_{7}) \end{aligned}$$

where $J_{1}$, $J_{2}$, $J_{4}$, $J_{5}$, $J_{6}$, and $J_{7}$ are the invariants characterizing the material symmetry in linear elasticity, that are defined as follows:

9 $$\begin{array}{*{20}l} {J_{1} = {\text{tr}}(\varvec{E}),} \hfill & {J_{2} = {\text{tr}}(\varvec{E}^{2} )} \hfill \\ {J_{4} {\text{ = }}\varvec{A}_{1} \cdot \varvec{EA}_{1} } \hfill & {J_{5} {\text{ = }}\varvec{A}_{1} \cdot \varvec{E}^{2} \varvec{A}_{1} } \hfill \\ {J_{6} {\text{ = }}\varvec{A}_{2} \cdot \varvec{EA}_{2} } \hfill & {J_{7} {\text{ = }}\varvec{A}_{2} \cdot \varvec{E}^{2} \varvec{A}_{2} } \hfill \\ \end{array}$$

Assuming small deformations, the dependence of the strain energy given by (8) could be assumed quadratic in the displacement field at leading order, and therefore, a function possessing material orthotropic symmetry is (Spencer 1982, 1984, Spencer and Soldatos 2007)

10 $$\begin{aligned} w_{1}(\varvec{E})&=\frac{1}{2}K\,{J_{1}}^{2}+\mu \, \left( J_{2}-\frac{1}{2}{J_{1}}^{2}\right) \nonumber \\&\quad +\left( \alpha _{1}\,J_{4} +\alpha _{2}\,J_{6}\right) \,J_{1}+2\mu _{1}\,J_{5}+2\mu _{2}\,J_{7}\\&\quad +\frac{1}{2}\beta _{1}\,{J_{4}}^{2}+\frac{1}{2}\beta _{2}\,{J_{6}}^{2} +\beta _{3}\,J_{4}J_{6}\nonumber \end{aligned}$$

in which *K*, $\mu$, $\mu _{1}$, $\mu _{2}$, and $\beta _{1}$, $\beta _{2}$ are material coefficients and $\alpha _{1}$, $\alpha _{2}$, and $\beta _{3}$ are coupling coefficients. We remark that the first two terms in (10) are associated with the hydrostatic and deviatoric contribution to the isotropic part of deformation. Here, all the material parameters may be inhomogeneous.

**Fig. 1:**
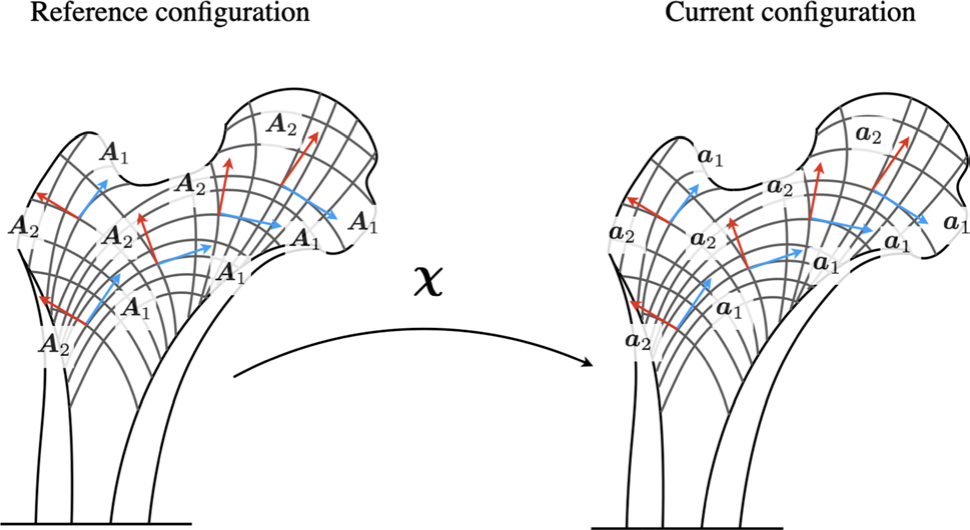
Material directions $\varvec{A}_{i}$ in the reference configuration aligned to the trabecula pattern and their images $\varvec{a}_{i}$ under the tensor $\varvec{F}$ for the section of a femur

To complete the definition of the elastic stored energy, we assume also a second gradient contribution. The reason for which we postulate this further contribution to the stored energy must be attributed to the trabecular substructure of bone. Indeed, in the regions characterized by a very low mass density, we can assume that the trabeculae are very thin and long with weak connections, which may imply the need, at macro level, of second gradient models (Giorgio et al. 2017; Abdoul-Anziz and Seppecher 2018; Abdoul-Anziz et al. 2019; dell’Isola et al. 2022). This typically occurs in areas subject to minimal deformation (i.e., high porosity) or in the case of a disease like osteoporosis. In this special cases, a simple energy function which involves some second gradient effects with orthotropic symmetry is (Steigmann and dell’Isola 2015, Giorgio et al. 2016)

11 $$\begin{aligned} w_{2}(\nabla \varvec{E})=\frac{1}{2}\left( \mathbb {K}_{1}\, \vert (\varvec{A}_{1})_{p}(\varvec{A}_{1})_{q}\, \varvec{\chi }_{,pq}\vert ^{2}+\mathbb {K}_{2}\, \vert (\varvec{A}_{2})_{p}(\varvec{A}_{2})_{q}\, \varvec{\chi }_{,pq}\vert ^{2}\right) \end{aligned}$$

where $\mathbb {K}_{1}$ and $\mathbb {K}_{2}$ are second gradient moduli and the notation “,” indicates partial differentiation. Moreover, the second gradient contributions can be split into the tangential stretch gradient and the curvature parts considering the decomposition:

12 $$\begin{aligned} (\varvec{A}_{i})_{p}(\varvec{A}_{i})_{q}\, \varvec{\chi }_{,pq}=(\varvec{A}_{i}\cdot \nabla \lambda _{i})\, \varvec{a}_{i}+\lambda _{i}^{2}\eta _{i}\varvec{n}_{i} \end{aligned}$$

where $\lambda _{i}\varvec{a}_{i}=\varvec{FA}_{i}$ (no summation convention), $\lambda _{i}=\left| \varvec{FA}_{i}\right|$, $\eta _{i}$ are the geodesic curvatures induced by the deformation on the trabecula (Shirani and Steigmann 2020) (see, e.g., Fig. 1). The vectors $\varvec{n}_{i}$ are orthogonal to $\varvec{a}_{i}$. With the decomposition (12), the second gradient energy density is assumed to be

13 $$\begin{aligned} w_{2}(\nabla \varvec{E})=\, & {} \frac{1}{2}\left[ \mathbb {K}_{1}^{s}\, (\varvec{A}_{1}\cdot \nabla \lambda _{1})^{2}+\mathbb {K}_{2}^{s}\, (\varvec{A}_{2}\cdot \nabla \lambda _{2})^{2}\right] \nonumber \\{} & {} +\frac{1}{2}\left[ \mathbb {K}_{1}^{b}\,\left( \lambda _{1}^{2}\eta _{1} \right) ^{2}+\mathbb {K}_{2}^{b}\,\left( \lambda _{2}^{2}\eta _{2} \right) ^{2}\right] \end{aligned}$$

being explicit the terms which refer to tangential stretch gradient effects and bending deformation. The same splitting procedure can be made for the stiffnesses, so we have $\mathbb {K}_{1}^{s}$ and $\mathbb {K}_{2}^{s}$ for the tangential stretch gradient stiffnesses and $\mathbb {K}_{1}^{b}$ and $\mathbb {K}_{2}^{b}$ for the bending stiffnesses.

The mechano-biologic energy density, $w_{r}$, responsible for the remodeling is considered as the summation of several contributions. Particularly, one, $w_{R}$, is related to the reorientation of the orthotropic symmetry direction $\varvec{A}_{1}$ (which is associated with the directions of trabecular substructure), and all the others gathered in $w_{s}$ are associated with the evolution of the material stiffnesses characterizing the mechanical response. Thus, we have:

14 $$\begin{aligned} w_{r}(\gamma ,\varvec{p},\varvec{S})=w_{R}(\gamma ) +w_{s}(\varvec{p},\varvec{S}) \end{aligned}$$

By specifying the stored strain energy as (10) and (11), we can specialize $\varvec{p}$ as $\{p_{1},p_{2},\dots ,p_{13}\}=\{K,\mu ,\alpha _{1},\alpha _{2},\dots ,\mathbb {K}_{2}^{b}\}$.

With the definition (4), we assume that

15 $$\begin{aligned} w_{R}(\gamma )=\frac{1}{2}\tau _{\gamma }\gamma ^{2} \end{aligned}$$

where $\tau _{\gamma }$ is a mechano-biological parameter associated with the level of energy that can be stored in such a process.

It is worth mentioning that, in a more general context, the angle $\psi$ can be replaced with the rotation $\varvec{Q}$ and the relative angle can be evaluated approximately by

16 $$\begin{aligned} \varvec{\Gamma }=\varvec{Q}^{\top }\varvec{F} \end{aligned}$$

since the deformation tensor $\varvec{U}$ tends to ${\textbf{I}}$ in the small deformation regime.^1^ Therefore, we have

17 $$\begin{aligned} \gamma \approx \text {arctan}\left(\frac{\Gamma _{21}}{\Gamma _{11}}\right) \end{aligned}$$

In the mechano-biological process, some energy dissipation occurs over time during the transition between different orientations; therefore, we define a Rayleigh dissipation function also to take into account this aspect, namely

18 $$\begin{aligned} {\mathscr {D}}_{R}=\frac{1}{2}c_{\gamma }{\dot{\gamma }}^{2} \end{aligned}$$

where $c_{\gamma }$ is a kind of “viscous” parameter. Therefore, a term $c_{\gamma }{\dot{\gamma }}\,\delta \gamma$ can be introduced in the virtual-work formulation, which implies a non-conservative phenomenon.

To define the evolution of all the material stiffnesses and coupling coefficients, we use the contribution $w_{s}$ which depends on them. In particular, for each material parameter $p_{i}$ (with $i=1,\dots ,13$), we can define the biologic potential energy as

19 $$\begin{aligned} {w_{s}}_{i}(p_{i},S_{p_{i}})=-{\mathscr {A}}(S_{p_{i}})\,p_{i} \end{aligned}$$

where ${\mathscr {A}}(S_{p_{i}})$ can be interpreted as a *remodeling action* which affects the material parameter $p_{i}$ and $S_{p_{i}}$ is the stimulus associated to such an evolution (Giorgio et al. 2019). The remodeling action is assumed to be a linear function of the stimulus as follows:

20 $$\begin{aligned} {\mathscr {A}}(S_{p_{i}})=\tau _{p_{i}}(S_{p_{i}}-S_{p_{i}}^{0}) \end{aligned}$$

where $\tau _{p_{i}}$ represents a mechano-biological gain to be tuned with experimental evidence and $S_{p_{i}}^{0}$ is a reference stimulus introduced to set the homeostatic state. The difference between the actual stimulus $S_{p_{i}}$ and the reference one gives feedback on how the system is far from homeostasis and, therefore, drives the evolution to restore this functional point of operation.

Finally, gathering all the contributions for the stiffnesses, we postulate

21 $$\begin{aligned} w_{s}=\sum _{i}{w_{s}}_{i}(p_{i},S_{p_{i}}) \end{aligned}$$

Similarly to the reorientation evolution, a Rayleigh dissipation function is introduced also for the materials coefficients $p_{i}$ in the form:

22 $$\begin{aligned} {{\mathscr {D}}_{s}}_{i}=\frac{1}{2}c_{p_{i}}\,\dot{p_{i}}^{2} \end{aligned}$$

with $c_{p_{i}}$ the related viscous coefficient.

### Evolution equations for *stimuli*

In this section, we postulate, independently from the principle of virtual work, a set of evolution equations for bio-mechanical stimuli.

We consider as an open, and important, problem the postulation of a unique variational principle from which this last kind of evolution equations can be deduced as consequence (see, Barchiesi and Hamila 2022, for a first attempt, even if in a slightly different context). Here, we consider the stimulus field (as depending, in our simplified model, on the material particle and time) as a modeling tool in the description of the mechanically guided remodeling action occurring in bone tissues.

We postulate that the mechanical state, acquired by sensor cells, i.e., osteocytes, determines this stimulus. This stimulus is then the activator of the biological agents which remodel the bone tissue. As the principal path for the transmission of the information acquired by sensor cells, concerns the action of paracrine factors, which are chemical agents, released and spread via the diffusion, we postulate a diffusive model to describe the evolution of the stimulus field.

Therefore, for each material parameter $p_{i}$, we postulate the evolution of the corresponding stimulus as follows:

23 $$\begin{aligned} d_{p_{i}}\frac{\partial S_{p_{i}}}{\partial t}=\nabla \cdot \left( \kappa _{p_{i}}\nabla S_{p_{i}}\right) +r(U_{p_{i}})-s(S_{p_{i}}) \end{aligned}$$

where $d_{p_{i}}$ is a damping coefficient, $\kappa _{p_{i}}$ the diffusion coefficient assumed constant and thus isotropic for the sake of definiteness and tractability, $r(U_{p_{i}})$ is a source term, and $s(S_{p_{i}})$ is an absorption function. Following the work (Branecka et al. 2022), where the evolution of different material coefficients is determined by the energy contribution associated with the same coefficient, here we set the source term *r* as proportional to the portion of the energy density relative to the same coefficient. For example, to define this contribution related to the shear coefficient $\mu$ in (10), we set in the bi-dimensional formulation

24 $$\begin{aligned} r(U_{\mu })=\mu \,\left( J_{2}-\frac{1}{2}{J_{1}}^{2}\right) \end{aligned}$$

and, for the sake of simplicity, the coefficient of proportionality is assumed to be 1. As usually done in the diffusion equation, the sink term *s* is defined proportional to the stimulus itself as follows:

25 $$\begin{aligned} s(S_{p_{i}})=R\,S_{p_{i}} \end{aligned}$$

being *R* the absorption coefficient related to the metabolic activity associated with the stimulus.

## Numerical results and discussion

**Fig. 2:**
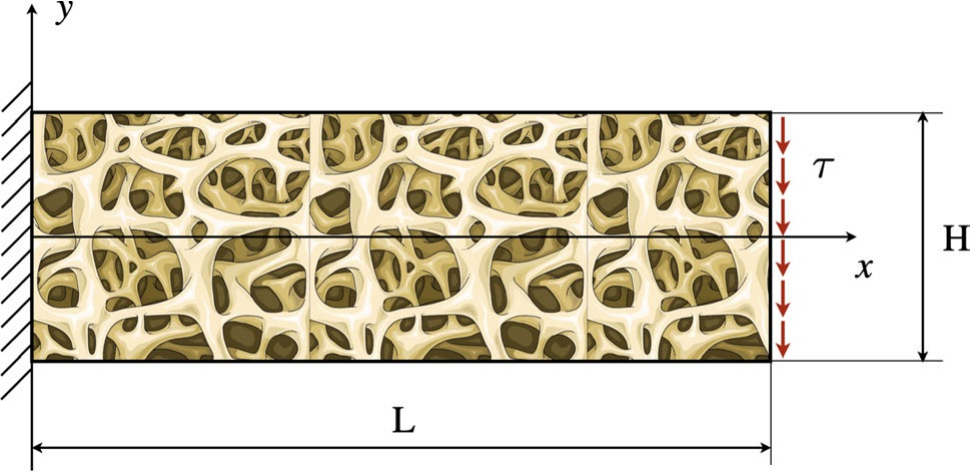
Cantilever plate bending test set-up

The proposed model has been tested through numerical simulations in some illustrative cases. In particular, we consider a rectangular sample made of trabecular bone tissue. In the first test, we analyze a cantilever plate under a uniform distribution of a shear force density $\tau (t)$ on the side opposite the clamp (see Fig. 2). The second test, instead, is a three-point flexural one, where we employ the symmetry of the system to examine only half of the specimen (see Fig. 10). Since our formulation is variational, the numerical implementation of the tests has been conducted with the finite element method using the principle of virtual work (5) directly inside the commercial software COMSOL Multiphysics. We perform simulations in Comsol for testing the proposed approach because of its high flexibility and ease of use. Indeed, it is possible to implement the governing equation directly using the weak formulation. However, the general-purpose nature of the software does not allow for specific code optimization for the considered problem. To achieve this, one can consider implementing isogeometric analysis (Greco and Cuomo 2021; Greco et al. 2021; Torabi and Niiranen 2023) or an ad hoc discretization (Turco et al. 2016; Turco 2018, 2022) in a subsequent phase of the development of the model.

The simulations has been performed for a rectangular domain of $75\times 25$ mm dimension, and the initial material stiffnesses are listed in Table 1. The second gradient stiffnesses, for the sake of simplicity, are set to be equal, namely $\mathbb {K}_{1}^{s}=\mathbb {K}_{2}^{s}=\mathbb {K}_{1}^{b}=\mathbb {K}_{2}^{b}=1.5$ N. The coefficients related to the evolutionary process for the reorientation of the material symmetry directions and the stiffnesses are reported in Tables 2 and 3. Herein, for the sake of simplicity and tractability, we postulate that the evolution of a few stiffnesses is more significant than the others, and then, we neglect the adaptive process of these last that changes their magnitude. Therefore, the only stiffnesses we consider to evolve are the shear modulus $\mu$ and the stretching stiffnesses in the material symmetry directions, i.e., $\beta _{1}$ and $\beta _{2}$. Finally, Table 4 shows the coefficients employed in the diffusion equations of the stimuli corresponding to the stiffnesses that are assumed to adapt to external mechanical conditions. The initial values of the stimuli are set to zero and Neumann boundary conditions specify that the stimuli flow vanishes to have insulation from the outside. In what follows, on the boundaries for which the boundary condition is not specified, the weak contribution or the dual is set to zero, as typically done for the adopted formulation.

**Table 1:** Initial material parameters (GPa)

*K*	$\mu$	$\alpha _{1}$	$\alpha _{2}$	$\mu _{1}$	$\mu _{2}$	$\beta _{1}$	$\beta _{2}$	$\beta _{3}$
17.84	7.32	$-2.87$	$-2.25$	$-1.71$	$-1.09$	2.97	1.47	2.14

**Table 2:** Evolutionary parameters for the reorientation process

$c_{\gamma }$	$\tau {}_{\gamma }$
$6.048\times 10^{5} \,\hbox {Pa}\,\hbox {s}$	$1\times 10^{-3}\,\hbox {Pa}$

**Table 3:** Evolutionary parameters for the stiffness adaptive recalibration

$c_{\mu }$	$\tau _{\mu }$	$S_{\mu }^{0}$
$6.048\times 10^{12}\,\hbox {Pa}\,\hbox {s}$	$1\times 10^{4}\,\hbox {Pa}$	3660 Pa
$c_{\beta _{1}}$	$\tau _{\beta _{1}}$	$S_{\beta _{1}}^{0}$
$6.048\times 10^{12}\,\hbox {Pa}\,\hbox {s}$	$1\times 10^{4}\,\hbox {Pa}$	1484.8 Pa
$c_{\beta _{2}}$	$\tau _{\beta _{2}}$	$S_{\beta _{2}}^{0}$
$6.048\times 10^{12}\,\hbox {Pa}\,\hbox {s}$	$1\times 10^{4}\,\hbox {Pa}$	734.29 Pa

**Table 4:** Evolutionary parameters for the diffusion of the stimuli

$d_{\mu }$	$\kappa _{\mu }$	$R_{\mu }$
$3.024\times 10^{6}\,\hbox {Pa}\,\hbox {s}$	$1\times 10^{-4}$ N	$0.35\times 10^{2}\,\hbox {Pa}$
$d_{\beta _{1}}$	$\kappa _{\beta _{1}}$	$R_{\beta _{1}}$
$6.048\times 10^{6}\,\hbox {Pa}\,\hbox {s}$	$1\times 10^{-4}$ N	$0.35\times 10^{2}\,\hbox {Pa}$
$d_{\beta _{2}}$	$\kappa _{\beta _{2}}$	$R_{\beta _{2}}$
$6.048\times 10^{6}\,\hbox {Pa}\,\hbox {s}$	$1\times 10^{-4}$ N	$0.35\times 10^{2}\,\hbox {Pa}$

### A rectangular cantilever plate bending under a uniform load per unit length

In the first case, we perform a numerical simulation aiming at reproducing the behavior of a rectangular plate of bone tissue under the typical arrangement of a cantilever system. We set the displacement vector of the left short edge zero and apply a uniform shear force per unit length on the opposite edge variable in time. The initial transient of the force is designed to gradually reach a steady-state condition and avoid sudden variations of the displacement that could induce some relevant inertial effects. The expression of the force applied is as follows:

26 $$\begin{aligned} \tau (t)={\left\{ \begin{array}{ll} \tau _{0}\left[ \frac{t}{T_{s}}-\frac{1}{2\pi }\sin \left( 2\pi \frac{t}{T_{s}}\right) \right] &{} t<T_{s}\\ \tau _{0} &{} t\geqslant T_{s} \end{array}\right. } \end{aligned}$$

where $\tau _{0}$ is the magnitude of the force and $T_{s}$ the extension of the transient. In particular, $T_{s} = 60,480 \,\hbox {s}$ and $\tau _{0}=1.25\times 10^{5}\,\hbox {N/m}$. This value corresponds to a steady-state condition that is reached for the strain energy density $w_{m}$ in the considered case.

**Fig. 3:**
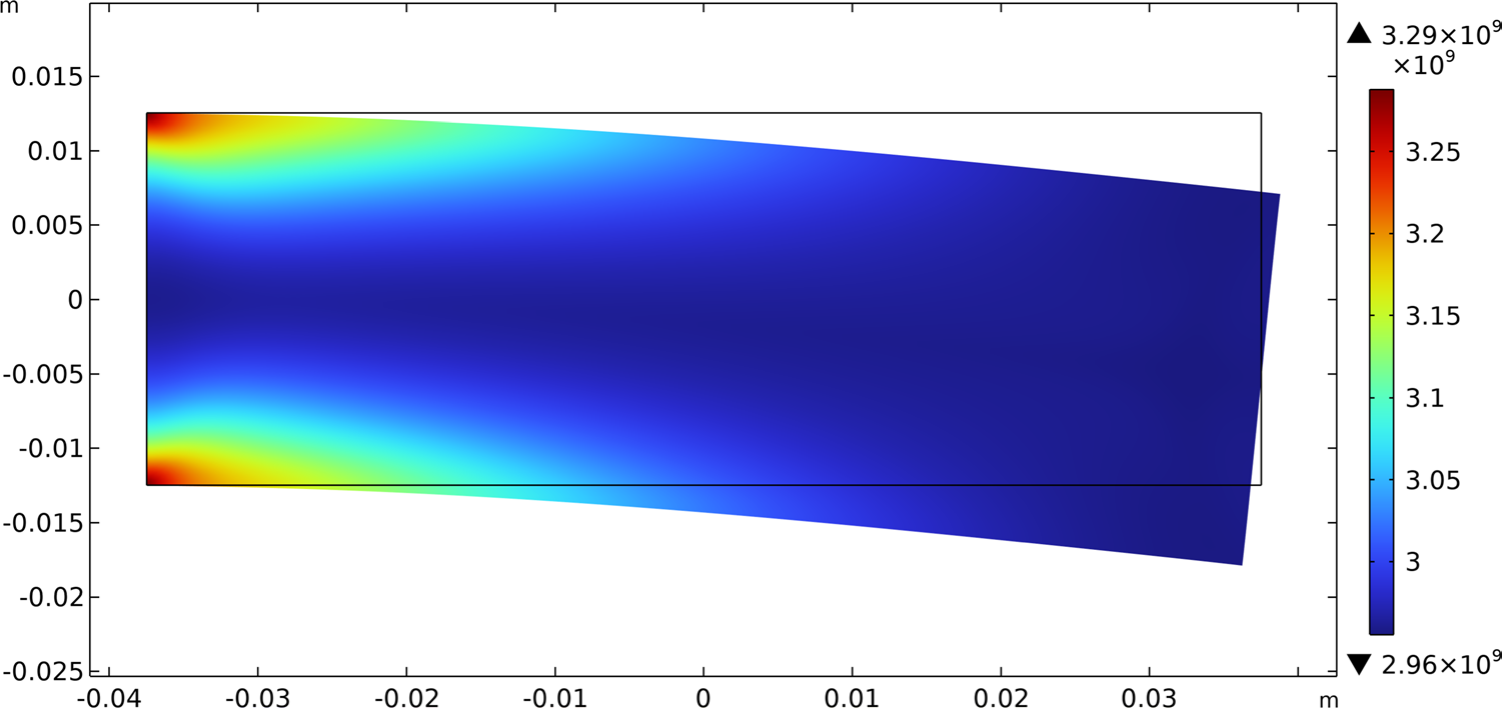
Bending test: distribution of $\beta _{1}$ at steady state after the application of the load

**Fig. 4:**
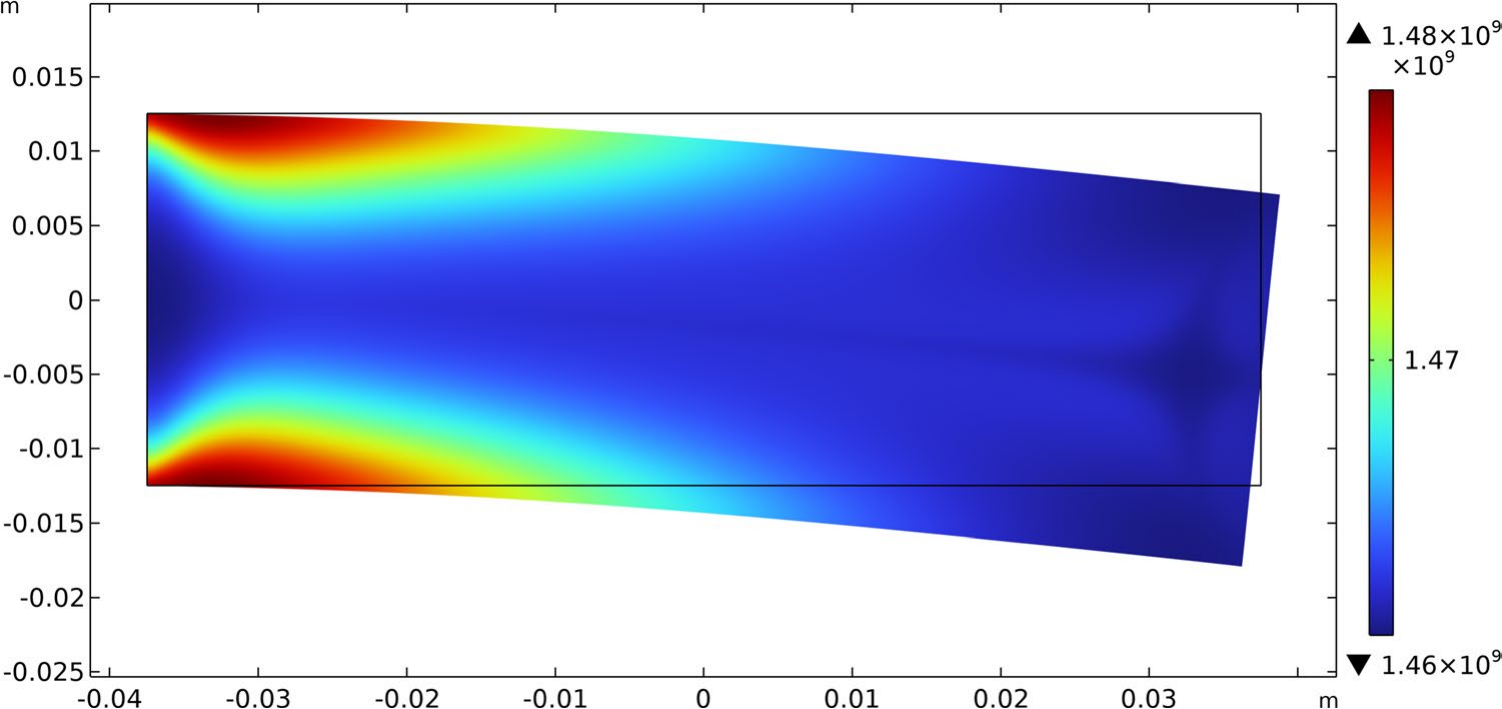
Bending test: distribution of $\beta _{2}$ at steady state after the application of the load

**Fig. 5:**
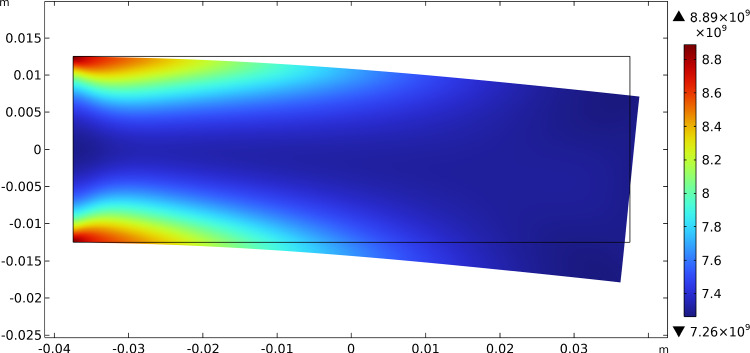
Bending test: distribution of $\mu$ at steady state after the application of the load

The results of this test show that the initially uniform distributed stiffnesses tend to increase their value where needed, i.e., where there is a localization of the contribution of energy associated with them, and decrease where the same energy density drops. Figures 3, 4, and 5 report this evolutionary change in the distribution of $\beta _{1}$, $\beta _{2}$, and $\mu$, respectively, together with the current final deformation. Indeed, the localization of the apposition of new bone tissue and its resorption is placed in the expected regions.

**Fig. 6:**
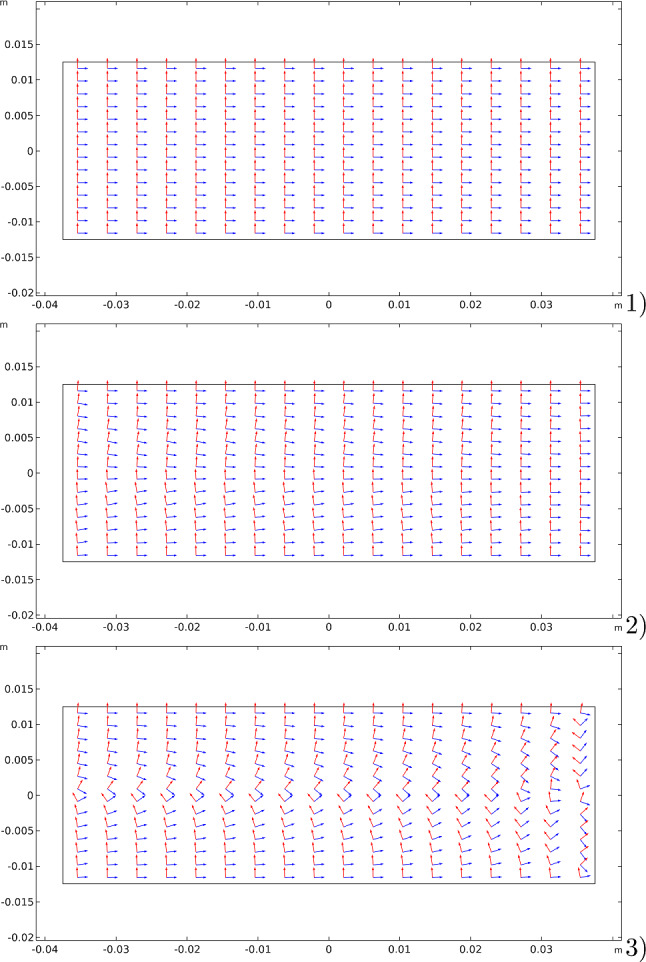
Bending test: evolution of the material symmetry directions for the bending cantilever test: (1) at the beginning; (2) at an intermediate stage; (3) at the steady-state condition

The other significant aspect of the proposed model, which is the possibility of having an evolution of the material symmetry directions, is shown in three-time subsequent steps (see Fig. 6): 1) at the beginning, where they are assumed parallel to the sides of the rectangular specimen; 2) at an intermediate step; and at the steady-state condition.

**Fig. 7:**
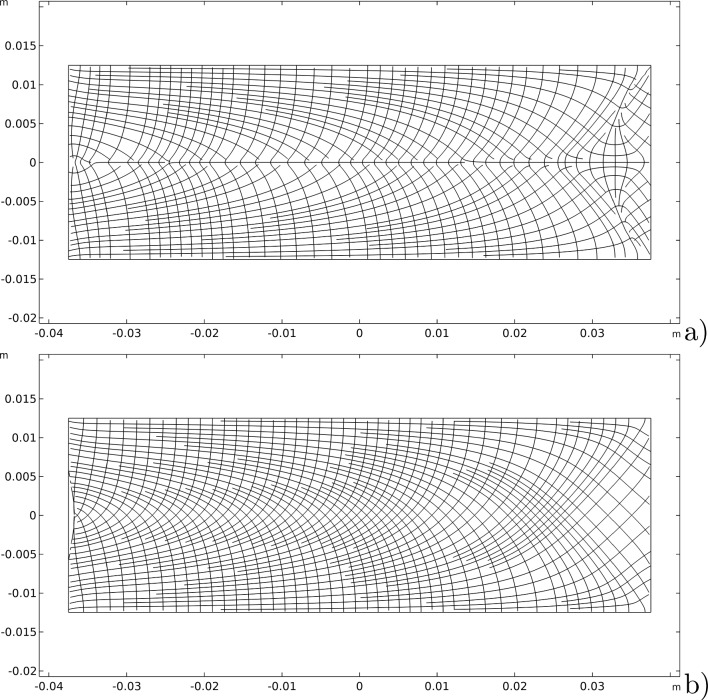
Comparison between: **a** streamlines generated by the unit vectors $\varvec{A}_{1}$ and $\varvec{A}_{2}$; b) isostatic lines for the bending test

To better understand the magnitude of this result, we consider the streamlines generated by these material symmetry directions at the end of the process and compare them with the isostatic lines related to the eigendirections of the strain tensor as shown in Fig. 7.

**Fig. 8:**
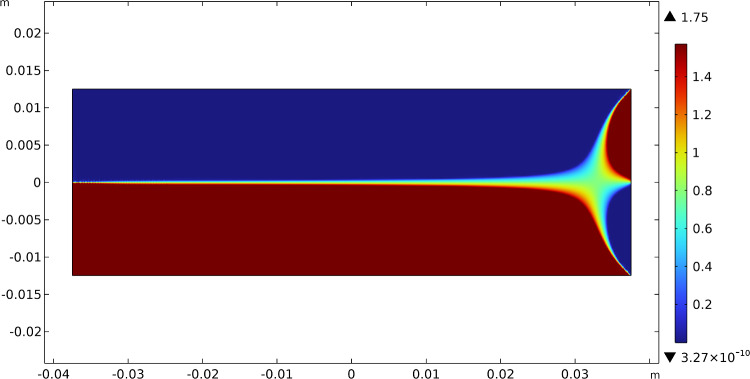
Bending test: difference between the angles between the vectors $\varvec{A}_{1}$ and $\varvec{A}_{2}$ and the eigenvectors of the stress

At first sight, they seem slightly different, but a more quantitative careful comparison demonstrates that they are the same almost everywhere except for very tiny regions (the green ones), as shown in Fig. 8. Actually, Fig. 8 displays the difference between the angles related to the vectors $\varvec{A}_{1}$ and $\varvec{A}_{2}$ and those associated with the eigenvectors of the strain. This plot allows seeing that these two fields share the same angle or an angle of $\pi /2$ in most of the domain. Therefore, since the two eigenvectors and the two fields of material symmetry must be orthogonal to each other, this implies that the isostatic and material symmetry directions coincide, and at least they exchange their order where the difference is $\pi /2$, accordingly with the trajectorial theory.

**Fig. 9:**
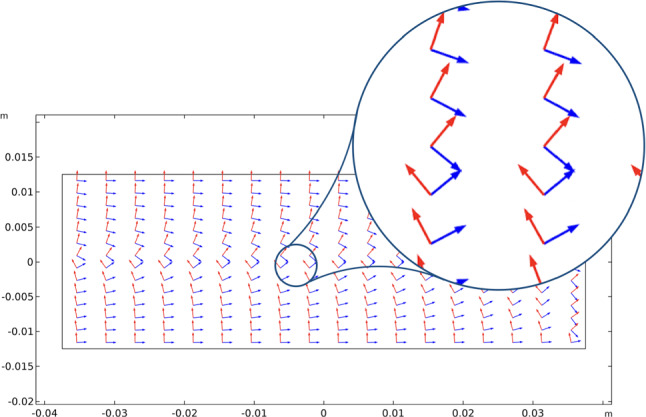
Bending test: Enlargement of the vectors $\varvec{A}_{1}$ (blue arrows) and $\varvec{A}_{2}$ (red arrows) in the transition zone

The slight changes in the angles in the green regions of Fig. 8 are indeed related to the exchange between the two vector fields passing through areas with a different representation, namely a sort of transition zone. We can notice in Fig. 9 that passing through this region the same direction of orthotropy $\varvec{A}_{i}$ (in the picture $\varvec{A}_{1}$ is indicated with blue arrows and $\varvec{A}_{2}$ with red arrows) stops being parallel to one eigendirection of the strain tensor to align with the other, which is rotated of $\pi /2$ relatively to the first, and simultaneously the same switch happens to the other direction of orthotropic symmetry.

### A three-point flexure test

The second case examined is a three-point flexure test. The effects of a test fixture having pins interacting with the bone specimen during the test are simulated with an elastic barrier of potential that impedes the sample from overlapping with them. Specifically, we use a frictionless penalty method to mimic the contact between the pins and the bone tissue. The specimen is placed on two fixed supporting pins set apart with a specific span length equal to $11/6\,L=137.5$ mm. A third loading pin in the middle of the test sample is used to deform the bone tissue gradually descending from above with a given time law. Here, we use the same expression as the previous test, Eq. (26), replacing the amplitude of the shear force with the assigned displacement to the loading pin $u_{0}=2$ mm. Due to the symmetry of the problem, we consider just half of the rectangular bone piece imposing symmetry conditions on the displacement at the middle section where the load is applied, namely $u_{1}=0$.

**Fig. 10:**
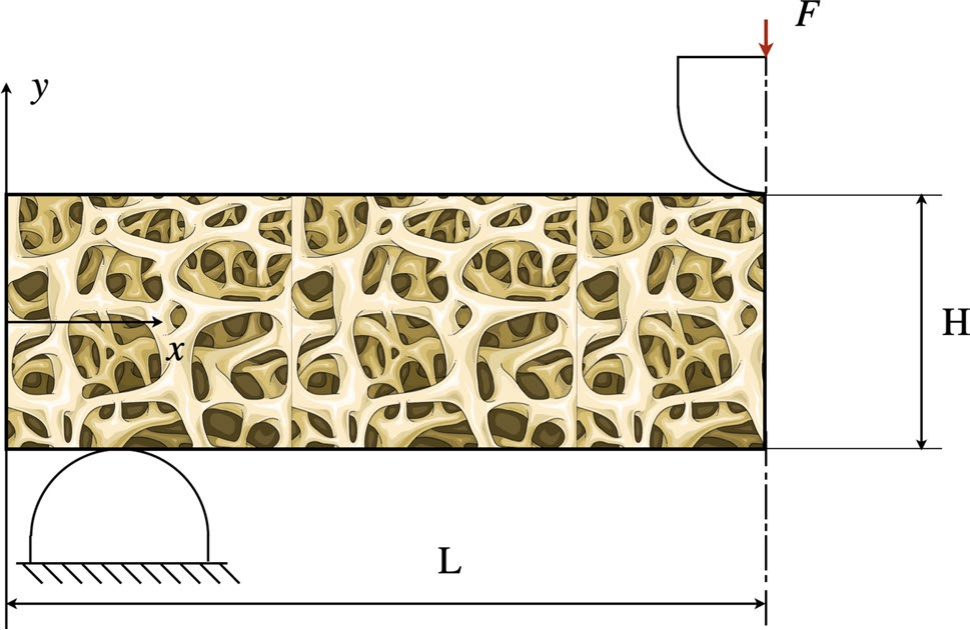
Three-point flexure test set-up

**Fig. 11:**
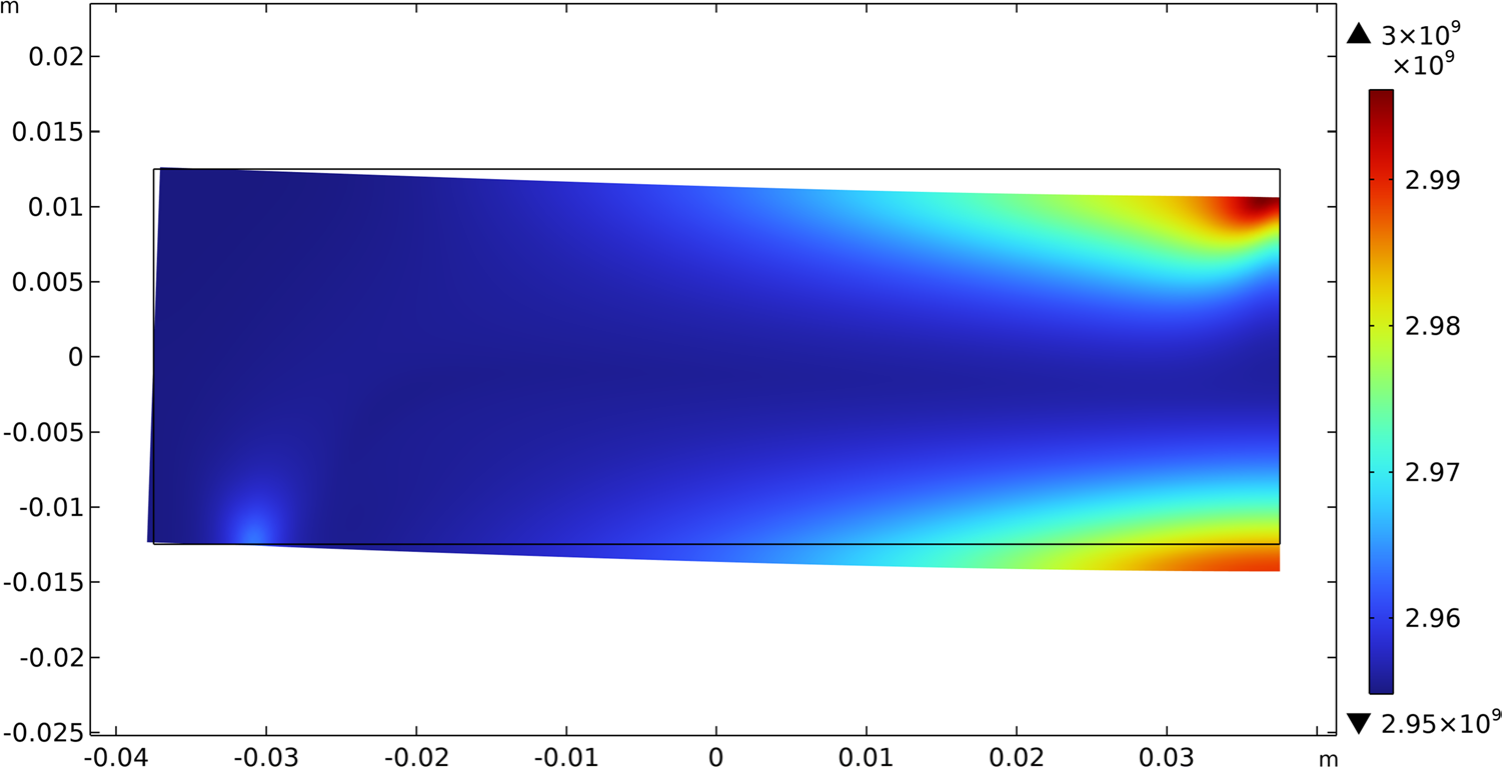
Three-point flexure test: distribution of $\beta _{1}$ at steady state after the application of the load

**Fig. 12:**
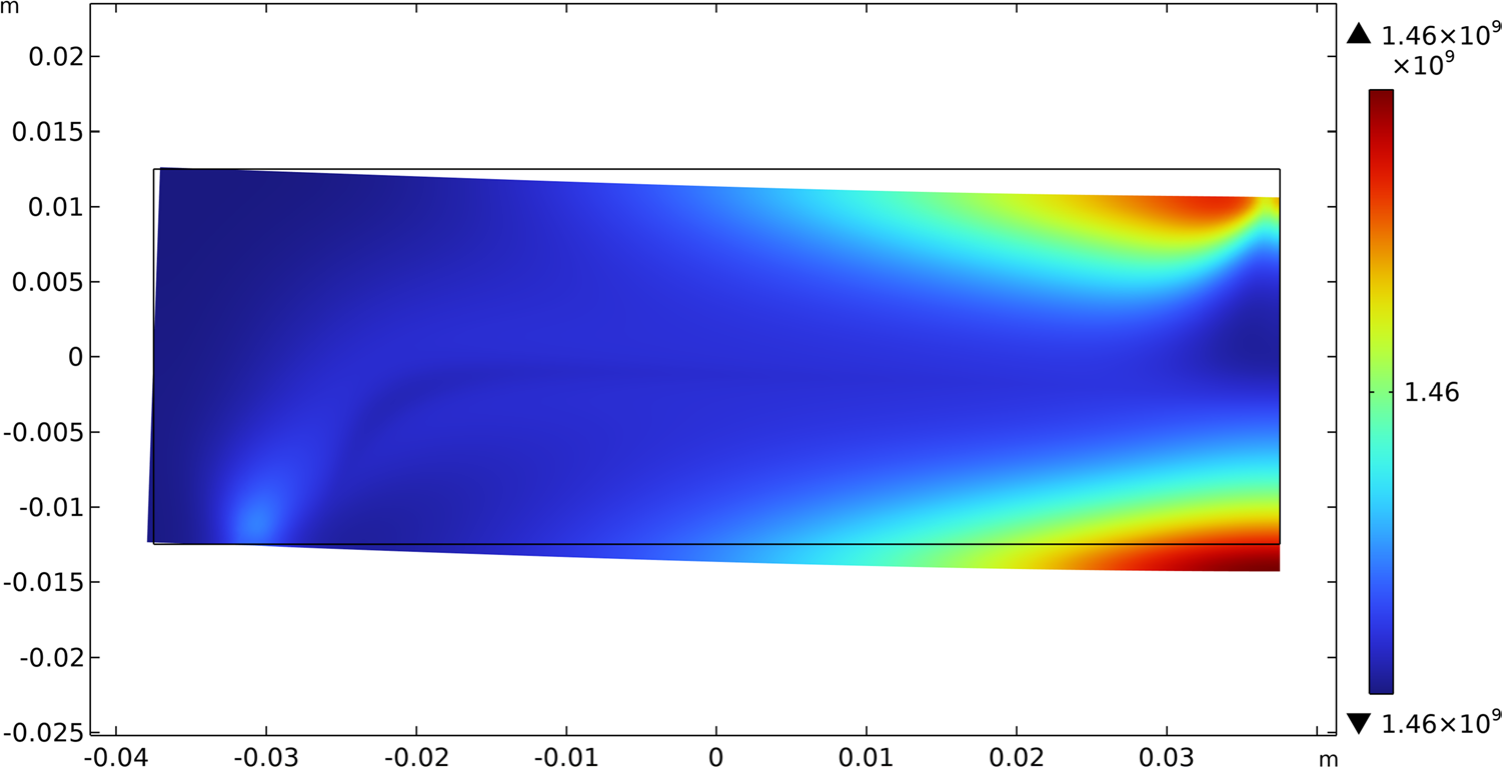
Three-point flexure test: distribution of $\beta _{2}$ at steady state after the application of the load

**Fig. 13:**
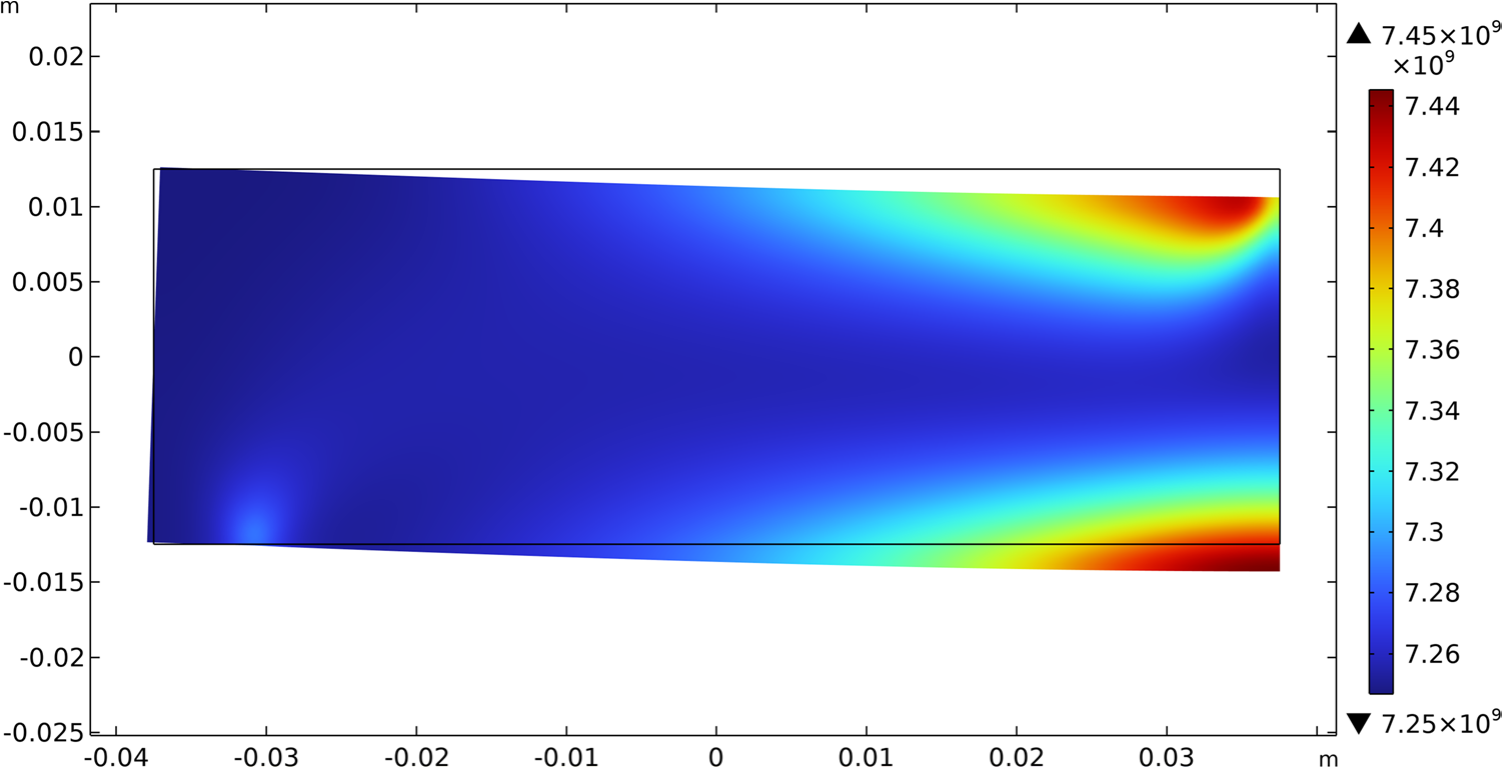
Three-point flexure test: distribution of $\mu$ at steady state after the application of the load

Figures 11, 12, and 13 exhibit the evolutionary change in the distribution of $\beta _{1}$, $\beta _{2}$, and $\mu$, respectively, together with the current final deformation. As expected, analogously to the previous case, a distribution of the stiffnesses reflecting the actual distribution of the energy density of the considered contributions is achieved.

**Fig. 14:**
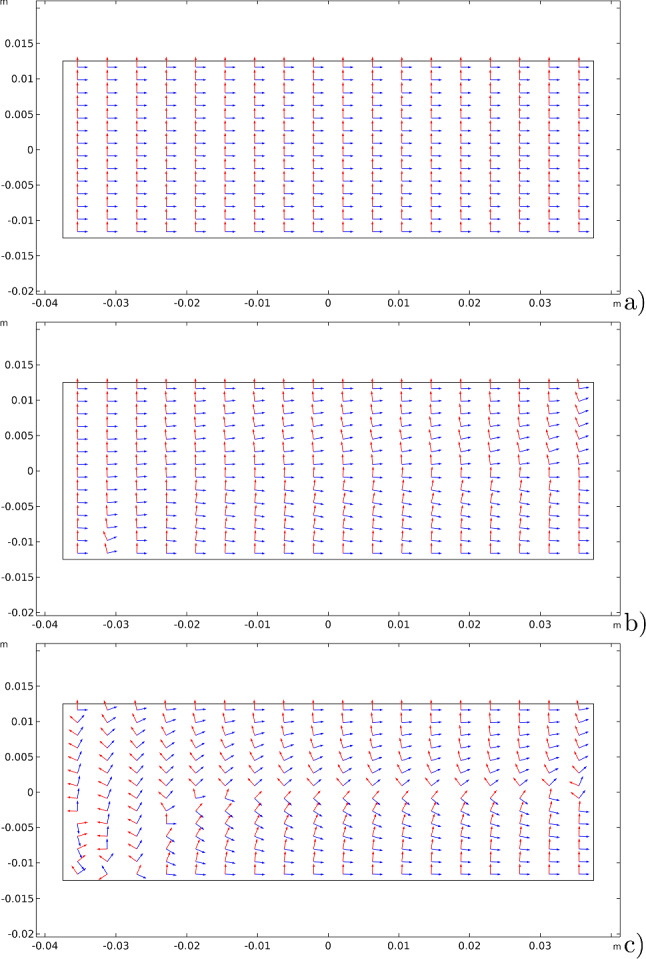
Three-point flexure test: evolution of the material symmetry directions for the bending cantilever test: **a** at the beginning; **b** at an intermediate stage; **c** at the steady-state condition

**Fig. 15:**
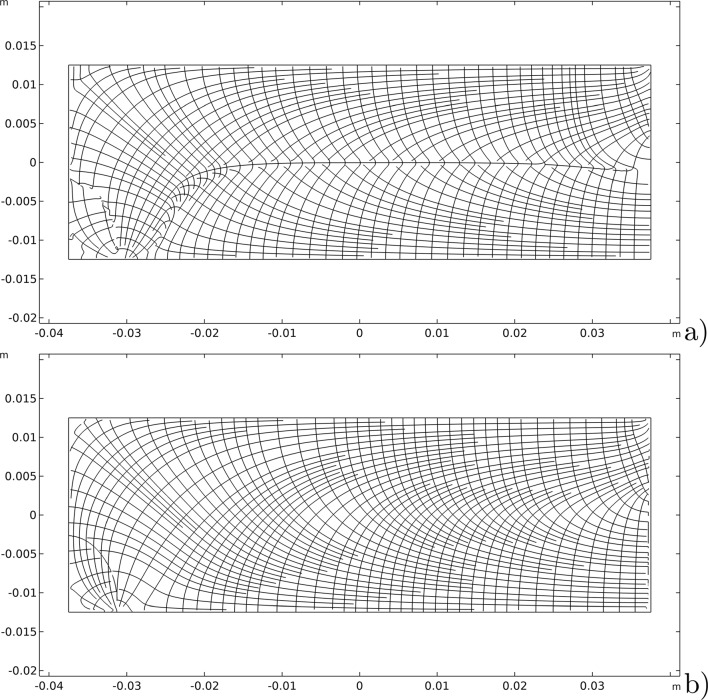
Comparison between: **a** streamlines generated by the unit vectors $\varvec{A}_{1}$ and $\varvec{A}_{2}$; **b** isostatic lines for the three-point flexure test

**Fig. 16:**
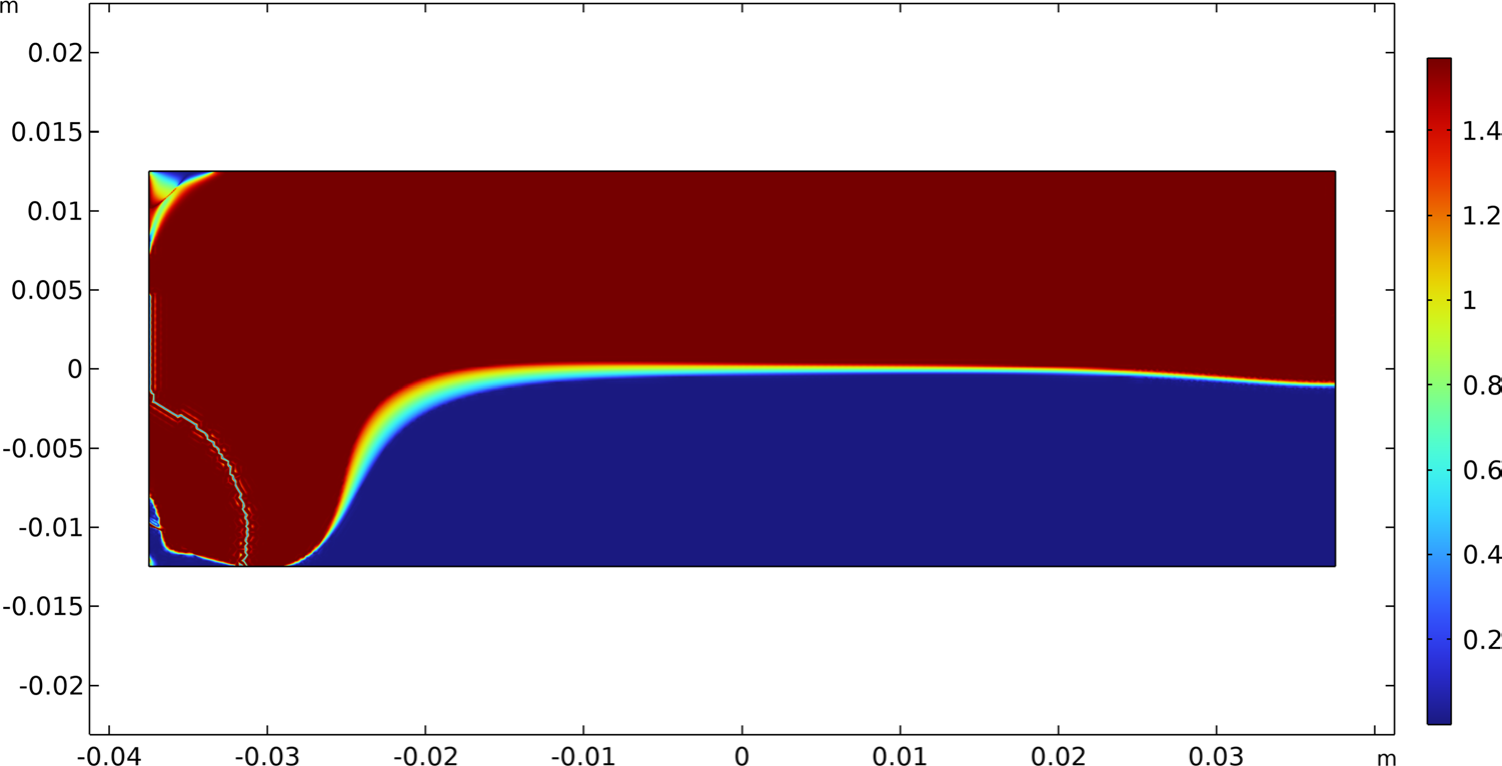
Three-point flexure test: difference between the angles between the vectors $\varvec{A}_{1}$ and $\varvec{A}_{2}$ and the eigenvectors of the stress

Figure 14 shows the evolutions of the material symmetry directions as the remodeling process progresses at three significant time instants. Their behavior is characterized by the alignment of them with the isostatic lines of the particular test considered, as can be clearly demonstrated by Fig. 15 where the streamlines generated by the unit vectors $\varvec{A}_{1}$ and $\varvec{A}_{2}$, as well as the isostatic lines, are displayed. Once again, their difference is very small and limited to narrow areas with a switch in the order that yields a difference of $\pi /2$, thus, a matter of representation, as it is quantified in Fig. 16.

## Conclusions

Bone remodeling is the process by which bones continually renew themselves throughout a person’s life. It involves the dissolution and absorption of old bone tissue by resorbing the mineralized matrix, breaking down the collagen fibers, and forming new bone tissue in a coordinated and controlled manner. Bone remodeling is essential for several reasons. It allows bones to repair themselves after injury or damage, helps to regulate the amount of calcium in the body, and plays a crucial role in maintaining bone strength and integrity. Besides, it also allows the bone to adapt to changes in stress and load over time since the new bone tissue is laid down in a highly organized manner and remodeled to meet the demands of the surrounding tissue. The process is influenced by a variety of factors, including hormones, diet, aging, and mainly physical activity. This process is really complex and we are far from completely understanding all its aspects. However, in this paper, we propose a new variational formulation that, by using the mechanical influence of the external load, is capable of explaining the adaptive process of rearranging the network of interconnected bone struts in the trabecular bone as well as their ability to carry on the load to which it is subject. The mechanical model is based on a generalized continuum theory and is characterized by an orthotopic material symmetry, which is entirely plausible for this material (Allena and Cluzel 2018; Cluzel and Allena 2018). The generalization involves some second gradient effects that could be relevant in specific contexts where the bone mass density is rarefied, and the lattice arrangement of the trabeculae is made of very thin and long struts with weak connections. Expressly, we assume a generalized principle of virtual work, assuming two key aspects, one related to the mechanical behavior of the bone tissue and the other concerning the evolutionary process that allows changing the orientations of the trabeculae as well as their thickness and length at the macroscopic level of observation of the entire sample. To illustrate the effectiveness of our proposal, two numerical tests have been performed. The results are promising and encouraging since they allow us to recover the optimization process typical of bone remodeling in the more general situation of orthotopic materials. Indeed, we are able to obtain not only a likely distribution of stiffnesses in the considered sample but also a reorientation of the material symmetry related to the actual arrangement of the trabeculae at a micro-level as postulated by the trajectorial theory for which we have experimental evidence.

## Footnotes

1 Indeed, we can write:

$$\begin{aligned} \varvec{\Gamma }= & {} \varvec{Q}^{\top }\varvec{R} \varvec{U}\approx \varvec{Q}^{\top }\varvec{R}=\left( \begin{array}{cc} \cos (\psi ) &{} \sin (\psi )\\ -\sin (\psi ) &{} \cos (\psi ) \end{array}\right) \left( \begin{array}{cc} \cos (\vartheta ) &{} -\sin (\vartheta )\\ \sin (\vartheta ) &{} \cos (\vartheta ) \end{array}\right) \\= & {} \left( \begin{array}{cc} \cos (\vartheta -\psi ) &{} -\sin (\vartheta -\psi )\\ \sin (\vartheta -\psi ) &{} \cos (\vartheta -\psi ) \end{array}\right) \end{aligned}$$

and, thus, an approximation of the relative angle $\gamma =\vartheta -\psi$.
